# Targeting Low-Phytate Soybean Genotypes Without Compromising Desirable Phosphorus-Acquisition Traits

**DOI:** 10.3389/fgene.2020.574547

**Published:** 2020-12-14

**Authors:** Mireadili Kuerban, Wenfeng Jiao, Jiayin Pang, Jingying Jing, Li-Juan Qiu, Wenli Ding, Wen-Feng Cong, Fusuo Zhang, Hans Lambers

**Affiliations:** ^1^College of Resources and Environmental Sciences, National Academy of Agriculture Green Development, Key Laboratory of Plant-Soil Interactions, Ministry of Education, China Agricultural University, Beijing, China; ^2^The UWA Institute of Agriculture, The University of Western Australia, Perth, WA, Australia; ^3^School of Agriculture and Environment, The University of Western Australia, Perth, WA, Australia; ^4^College of Grassland Science and Technology, China Agricultural University, Beijing, China; ^5^National Key Facility for Gene Resources and Genetic Improvement, Key Laboratory of Crop Germplasm Utilization, Ministry of Agriculture, Institute of Crop Sciences, Chinese Academy of Agricultural Sciences, Beijing, China; ^6^School of Biological Sciences, The University of Western Australia, Perth, WA, Australia

**Keywords:** soybean germplasm, phytate, phosphorus-acquisition efficiency, root morphology, carboxylates

## Abstract

Phytate-phosphorus (P) in food and feed is not efficiently utilized by humans and non-ruminant livestock, potentially contributing to high losses of P to the environment. Crops with high P-acquisition efficiency can access soil P effectively. It remains elusive whether crop genotypes with high P-acquisition efficiency can also have low seed phytate concentrations. A core collection of 256 soybean [*Glycine max* (L.) Merr.] genotypes from China with diverse genetic background were grown in the same environment and seeds were sampled to screen for seed phytate-P concentration. Some of these genotypes were also grown in a low-P soil in the glasshouse to measure root morphological and physiological traits related to P acquisition. Large genotypic variation was found in seed phytate-P concentration (0.69–5.49 mg P g^–1^ dry weight), total root length, root surface area, rhizosheath carboxylates, and acid phosphatase activity in rhizosheath soil. Geographically, seed phytate-P concentration was the highest for the genotypes from Hainan Province, whereas it was the lowest for the genotypes from Inner Mongolia. Seed phytate-P concentration showed no correlation with any desirable root traits associated with enhanced P acquisition. Two genotypes (Siliyuan and Diliuhuangdou-2) with both low phytate concentrations and highly desirable P-acquisition traits were identified. This is the first study to show that some soybean genotypes have extremely low seed phytate concentrations, combined with important root traits for efficient P acquisition, offering material for breeding genotypes with low seed phytate-P concentrations.

## Introduction

Phosphorus (P) deficiency is a major limiting factor for crop production worldwide owing to low soil P availability. It is readily sorbed by oxides and hydroxides of aluminium (Al) and iron (Fe) in acid soils, and precipitated by calcium (Ca) in alkaline soils (Hinsinger, 2001; Raghothama and Karthikeyan, 2005), leading to 70–90% of P applied as fertilizer becoming unavailable to most crop plants (Holford, 1997). The high rates of P-fertilizer application in crop production result not only in gradually diminishing phosphate rock reserves, which is associated with decreasing P-fertilizer quality, but also in environmental issues associated with off-site effects of P fertilizers (Cordell et al., 2009; Ghaffar et al., 2017). Therefore, it is essential to enhance P-acquisition efficiency by breeding highly P-efficient crop genotypes (Cong et al., 2020).

Soybean [*Glycine max* (L.) Merr.] as a widely cultivated grain legume, is an important source of protein and vegetable oil for human consumption, and also widely used in animal feed globally (Yuan et al., 2007; Liang et al., 2010). In addition, soybean has a pivotal ecological function in cropping system, e.g., nitrogen fixation (Salvagiotti et al., 2008), soil carbon sequestration (Cong et al., 2015), enhancing soil P availability (Xia et al., 2013), and decreasing soil-borne diseases (Gao et al., 2014) for themselves and the following crops. Approximately 60–80% of total P in soybean seed accumulates as phytate (myo-inositol 1, 2, 3, 4, 5, 6-hexakisphosphate, PA, or IP6) (Raboy et al., 1984). A high phytate concentration in seed is an undesirable trait, because it renders zinc (Zn) and other micro-nutrients unavailable for humans and livestock, contributing to malnutrition, especially Zn and iron (Fe) deficiency (Raboy, 2001; Perera et al., 2019). Moreover, phytate cannot be efficiently utilized by humans and non-ruminant animals, contributing to high losses of P to the environment. Thus, breeding for low-phytate soybean genotypes with high P-acquisition efficiency is a highly desirable sustainable strategy. It is one of the vital and preliminary steps for improving the desired traits in breeding programs that explore the genetic variation in germplasm (Perera et al., 2019). Hence, there is a need to study the genetic variation in seed phytate-P using a large set of soybean genotypes with diverse genetic backgrounds.

Identifying P-efficient crop genotypes is a sustainable and effective way to tighten the P cycle, thereby reducing P-fertilizer input, and mitigating the risks of pollution of ground- and surface water (Cong et al., 2020). Genotypic differences in nutrient-use efficiency are closely related to differences in efficiency of nutrient acquisition by roots (Marschner, 1998). Root traits such as root size, morphology, physiology, and mycorrhizal associations play a dominant role, both in P acquisition and in the exploration of a large soil volume, particularly under low P availability in soil (Marschner, 1998). Root morphological and physiological traits may respond to P deficiency (Zhou et al., 2016). Fernandez and Rubio (2015) reported higher specific root length and smaller average root diameter in soybean where P-acquisition efficiency increased under P deficiency. Root physiological traits, mainly root exudates such as carboxylates and phosphatase enzymes, may also increase plant P-acquisition efficiency (Dinkelaker et al., 1989; Pang et al., 2018). Earlier work showed genetic variability of root architecture in a core collection of soybean germplasm, with a shallow root architecture associated with higher P efficiency (Ao et al., 2010). However, knowledge of genotypic variation in root size, morphological and physiological traits is largely unknown for soybean. Therefore, there is an urgent need to investigate the variation in root size including root dry weight and rhizosheath soil dry weight, root morphological traits including root surface area and total root length, and root physiological traits including the total amount of carboxylates in the rhizosheath and acid phosphatase activity in a large set of soybean genotypes with diverse genetic backgrounds.

Previous studies compared the seed phytate-P concentration of soybean genotypes (Horner et al., 2005; Maharjan et al., 2019), and mainly focused on variation in seed phytate-P concentration among genotypes and its relationship with nutrients such as Fe, Zn, and calcium (Ca), and protein within a relatively small set of genotypes. Thus, there is a chance they missed identifying soybean genotypes with low seed phytate-P concentration. Wang et al. (2007) have identified a core collection of soybean germplasm, on average representing 81.5% of genetic variation. This collection provides us with a great opportunity to identify genotype with low seed phytate-P concentration and its relationship with desirable root traits related to P acquisition in soil with a low P availability. Identification of genotypes with highly desirable P-acquisition traits with low phytate-P concentrations has many benefits. First, lowering the seed phytate-P concentration would enhance the bio-availability of micro-nutrients such as Zn and Fe for humans and farm animals (Raboy, 2001), and thus improve the nutritional quality of soymeal. Increased nutritional quality of soymeal would go hand in hand with decreasing environmental pollution, especially eutrophication of aquatic environments (Sharpley et al., 2000; Brinch et al., 2002). Second, genotypes with desirable P-acquisition traits and low unavailable seed P concentrations require less P fertilization, and therefore reduce the cost for farmers and risk of P losses from the field (Cong et al., 2020).

The aims of the present study were (1) to investigate the variation in seed phytate-P concentration among 256 soybean genotypes and their geographically distributed differences; (2) to study the variation in root dry weight and rhizosheath soil dry weight, root morphological traits including total root length and surface area, as well as root physiological traits such as the amount of carboxylates and acid phosphatase activity in the rhizosheath among a subset of genotypes originally from the North China Plain (the main food producing region) under low-P condition; (3) to examine if soybean genotypes with desirable low phytate-P concentrations can simultaneous express desirable root traits associated with high P-acquisition efficiency, using a subset of genotypes. We hypothesized that (1) there is a large variation in seed phytate-P concentration and root size, morphological and physiological traits among soybean genotypes; (2) seed phytate-P concentration does not correlate with any of the measured desirable root traits related to P acquisition.

## Materials and Methods

### Plant Material and Growing Conditions

We grew 256 genotypes (Supplementary Table 1) from divergent provinces of China in a single field in Hainan Province (108°37′–111°03′ E, 18°10′–20°10′ N), China to measure seed phytate-P concentrations. Annual mean temperature is 22°C with a minimum and maximum temperature of 17 and 27°C, respectively. Annual rainfall ranges from 1000 to 2600 mm with an average annual rainfall of 1639 mm. Annual sunshine ranges from 1750 to 2650 h, and the total solar radiation is 4600–5800 MJ m^–2^ year^–1^. The soil is classified latosol. Information on these genotypes including Province of origin is described in Supplementary Table 1.

Of the 256 genotypes, 43 from five provinces in the Huang-Huai-Hai plain and Middle-Lower Yangtze plain were used to study six root traits that are associated with P acquisition (Supplementary Table 1). Those 43 genotypes are representative, because they include both ones that show the lowest and the highest phytate concentration, and the coefficient of variation (CV) is similar to that of the data set comprising 256 genotypes.

Pot experiment was conducted to determine root traits in a glasshouse at China Agricultural University, Beijing, with a day temperature of 25–30°C and a night temperature of 18–22°C. River sand and a soil with a low P availability were collected from Changping in the Beijing area. River sand was washed, and then both river sand and soil were air-dried and sieved (2-mm mesh size) to remove coarse fragments and macro-arthropods prior to potting. Soil and sand were sterilized by gamma irradiation (>25 K Gray gamma irradiation before potting. Each pot (85 cm × 85 mm × 180 mm) was filled with 1.2 kg mixture of sterilized washed river sand and soil with a low P availability (in a ratio of 3:7, w/w). The pot experiment followed a completed randomized design with soybean genotype as the main factor. Each of 43 genotypes was replicated in four pots. Seeds were surface sterilized with 10% (v/v) hydrogen peroxide for 3 min, then washed with deionized water and germinated in the dark on moist filter paper at 25/20°C (day/night). In each pot, we planted four seeds at 20 mm depth and inoculated with *Rhizobium leguminosarum* bv. NM353 (provided by the Culture Collection of China Agricultural University). Seedlings were thinned to one plant per pot at 7 days after sowing. Each pot was watered with deionized water to 60% field capacity by weighting every other day.

### Determination of Seed Phytate Concentration

Grains of each soybean genotype were collected at maturity, dried and ground to a fine powder. The powder was then passed through a 0.5 mm sieve. The phytate concentration was determined using the trichloroacetic acid method (Perera et al., 2019). Briefly, 4 g of seed powder was added to 50 ml of 30 g l^–1^ trichloroacetic acid and kept for 2 h for digestion, and then shaken for 30 min, and filtered using dry filter paper. Ten ml of supernatant was treated with 4 ml of ferric chloride solution (FeCl_3_) in a boiling water bath for 45 min. After cooling, samples were centrifuged at 1,710 × *g* for 10 min. The supernatant was removed, and 20–25 ml of 0.18 M trichloroacetic acid solution was added to wash the samples; after that, the samples were boiled in a water bath for 10 min twice. The precipitate was treated with 20 ml of water and 3 ml of 1.5 M NaOH in a boiling water bath for 30 min. After centrifugation, 5–10 ml of supernatant was treated with 3 ml of a mixture of di-acids (2 nitric acid + 1 perchloric acid) for digestion on an electric furnace at low temperature until there were white fumes. After cooling, samples were washed with 30 ml water several times; then, 3 ml of nitric acid solution and 10 ml of chromogenic agent were added. After 20 min, the absorbance was measured spectrophotometrically at 420 nm.

### Plant Harvest and Measurements

Plants grown in the glasshouse were harvested 45 days after sowing. Shoots were separated from the roots. In order to collect rhizosheath exudates, the method of Pearse et al. (2007) was modified. Briefly, at harvest, each pot was squeezed gently to allow dislodgement of the soil column and loosening of soil around the roots. The roots were shaken lightly to remove excess bulk soil; the soil and sand remaining attached to the roots was defined as rhizosheath soil (Pang et al., 2017). The root system was then transferred into a beaker containing 50 ml of 0.2 mM CaCl_2_ to avoid cell damage and gently shaken for 60 s to remove as much of the rhizosheath soil as possible. For the determination of acid phosphomonoesterase (APase) activity in the rhizosheath soil, a 0.5 ml subsample of the rhizosheath extract was transferred into a 2 ml centrifuge tube (Alvey et al., 2001). This solution was incubated at 30°C for 30 min after adding 0.4 ml of 200 mM sodium acetate and 0.1 ml of 150 mM *p*NPP. After incubation, 0.5 ml of 0.5 M NaOH was added and the solution was filtered. Then, the absorbance of this solution was measured spectrophotometrically at 405 nm, representing rhizosphere APase (Nuruzzaman et al., 2006; Lyu et al., 2016). For the analysis of carboxylates, 10 ml of supernatant was taken and three drops of concentrated phosphoric acid and a microbial inhibitor, Micropur (Sicheres Trinkwasser, Graz, Austria) were added, and filtered through a 0.22 μm syringe filter into high performance liquid chromatography (HPLC) vials according to Shen et al. (2003). HPLC samples were frozen at −20°C until analysis. The rhizosheath soil in the rhizosphere exudate solution after extraction of APase and carboxylates was kept in the greenhouse to allow evaporation of excess solution. The rhizosheath soil was oven-dried at 105°C for 72 h, and the dry weight was recorded.

After collection of rhizosheath exudates, roots were washed free of remaining soil, spread out on a transparent plastic tray, and root images were obtained by Epson Perfection V700 dual lens scanning system at a resolution of 600 dpi (dots per inch). Root images were analyzed for total root length and total root surface area using WinRHIZO software (Pro 2009b, Regent Instruments Inc., Quebec City, Canada). After analyzing root images, roots were oven-dried at 70°C for 72 h until constant weight to measure biomass.

### Classification of Soybean Genotypes

Soybean genotypes were classified into four categories as proposed by Bilal et al. (2018). These categories include (i) a low-phytate group with desirable root traits, (ii) a low-phytate group with undesirable root traits, (iii) a high-phytate group with desirable root traits, and (iv) a high phytate-P group with undesirable root traits. Low phytate-P refers to genotypes having seed phytate-P concentration lower than the mean seed phytate-P concentration, and desirable root traits means genotypes having more desirable root traits than the mean root traits and *vice versa*.

### Statistical Analyses

Analysis of variance was used to analyses the differences in seed phytate-P concentration between genotypes used in different agroecological regions using SPSS19. Tukey’s *post hoc* test was further used to examine the differences in case the effect was significant. Principal component analysis (PCA) of all plant traits comprising seed phytate-P concentration and root traits based on Pearson’s correlation matrix was conducted by Genstat (version 18.2, Genstat Procedure Library Release PL26.2, VSN International, Hemel Hempstead, United Kingdom, 2016) to investigate their correlations.

## Results

### Seed Phytate-P Concentration

**FIGURE 1:**
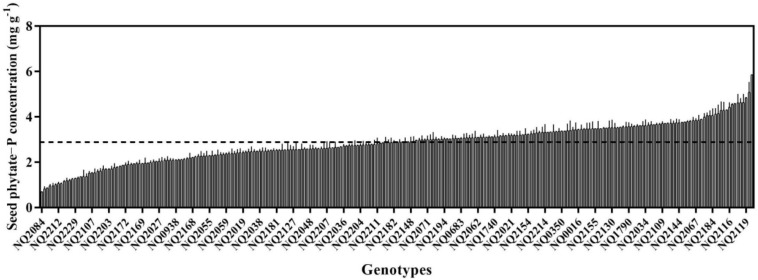
Seed phytate-P concentrations of 256 soybean genotypes, expressed in units of phosphorus (P). Data are means + SE (*N* = *2*). The dotted line represents the mean value of 256 soybean genotypes seed phytate-P concentration.

Seed phytate-P concentrations from plants grown in the same environment varied greatly, with approximately a seven-fold difference among 256 varieties, ranging from 0.69 mg P g^–1^ DW to 5.49 mg P g^–1^ DW, with a mean value of 2.88 mg P g^–1^ DW (Figure 1). Over half of the genotypes had a higher phytate concentration than the mean value. Two genotypes (Pixianalayinghuang and Heikewudou) had the highest seed phytate-P concentration (>5 mg g^–1^ DW), whereas five varieties (Dabaimaodou, Longchuanhuangniumao, Liushiribaidou, Siliyuan, and Lvcaodou) had very low values (<1 mg g^–1^ DW) (Figure 1).

### Geographic Background of Genotypes With Specific Seed Phytate-P Concentrations

**FIGURE 2:**
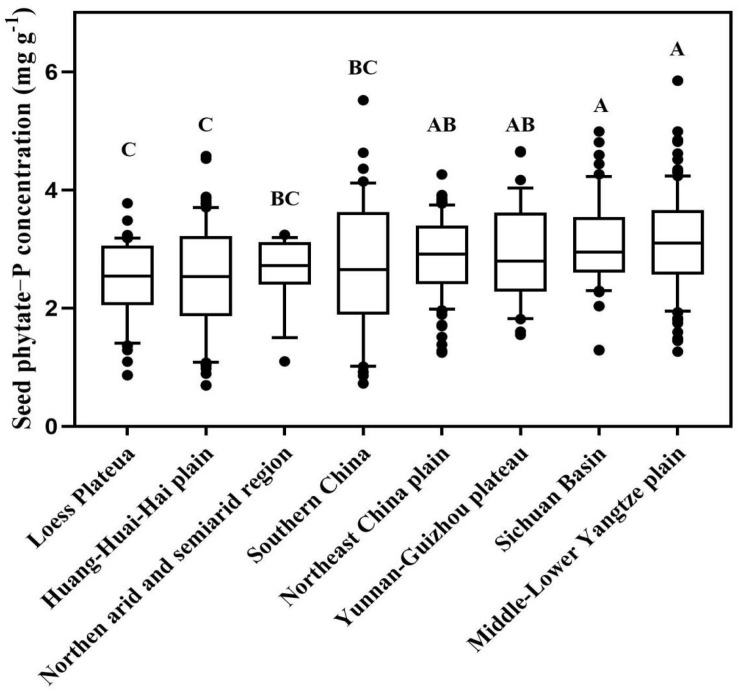
Spatial distribution characteristics of phytate-phosphorus (P) concentrations of 256 soybean genotypes among nine agroecological region. Each central vertical bar in box shows the mean, the box represents the inter-quartile range (IQR), the whiskers represent the location of the most extreme value points that are still within a factor of 10% of the upper or 90% lower quartile, and the black points are values that fall outside the whiskers. Different letters represent significant differences in seed phytate-P concentration among different agroecological regions (*P* < 0.05).

There was a large variation in seed phytate-P concentration among genotypes from 26 provinces (*P* < 0.05). Averaged for all provinces, the mean seed phytate concentration in the 26 provinces ranged from 2.31 mg P g^–1^ DW in Gansu to 5.08 mg P g^–1^ DW in Hainan Province, with a mean value of 2.94 mg P g^–1^ DW across China (Supplementary Figure 1 and Supplementary Table 2). The seed phytate-P concentration for genotypes from Hainan Province was 24∼54% greater than that in other provinces (*P* < 0.05). The coefficient of variation of seed phytate-P concentration in each Province ranged from 0 to 52%. Among nine agroecological region, seed phytate-P concentration ranged from 2.50 mg P g^–1^ DW on the Loess Plateau to 3.11 mg P g^–1^ DW on the Middle-Lower Yangtze Plain (*P* < 0.05, Figure 2).

### Root Traits

**FIGURE 3:**
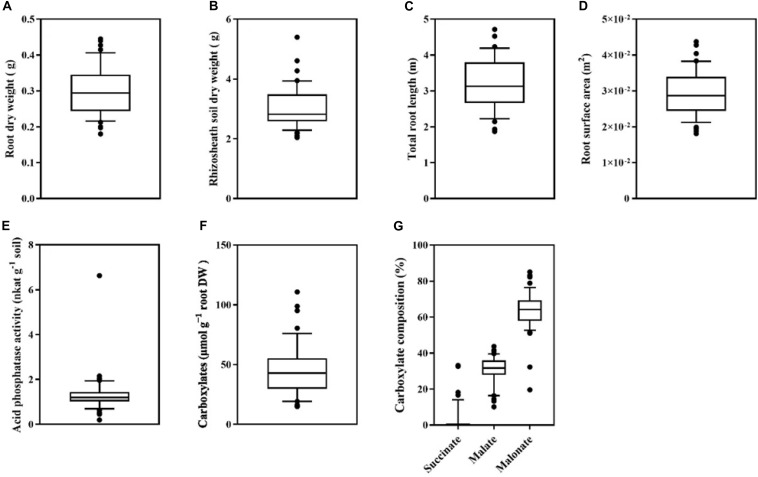
Box plots showing **(A)** root dry weight, **(B)** rhizosheath soil dry weight, **(C)** total root length, **(D)** root surface area, **(E)** acid phosphatase activity in the rhizosheath soil, **(F)** the amount of total carboxylates relative to root dry weight, **(G)** the composition of carboxylates in the rhizosheath soil consisting of succinate, malate, and malonate of 43 soybean genotypes grown for 45 days in washed river sand mixed with a low-phosphorus soil. Each black point represents the averaged value of four replicates. The central vertical bar in each box shows the mean, the box represents the inter-quartile range (IQR), the whiskers represent the location of the most extreme value points that are still within a factor of 10% of the upper or 90% lower quartile, and the black points are values that fall outside the whiskers.

There was a large variation among 43 genotypes in root dry weight (DW) and rhizosheath soil dry weight (*P* < 0.001 for both) (Figures 3A,B). Root dry weight varied 2.5-fold, ranging from 0.18 g DW plant^–1^ in Pingdingheito 0.45 g DW plant^–1^ in Dadunxiaoheidou (Figure 3A). Rhizosheath soil dry weight varied 2.7-fold, from 2.0 g DW plant^–1^ in Binhaidahuangkezijia to 5.4 g DW plant^–1^ in Siliyuan (Figure 3B).

An eight-fold difference in total root length was found among 43 soybean varieties, ranging from 0.77 to 6.88 m plant^–1^ (*P* < 0.001, Figure 3C). Similarly, root surface area also varied greatly among 43 soybean genotypes, ranging from 1.8 × 10^–2^ m^2^ plant^–1^ in Xichuanjiwohuang to 4.4 × 10^–2^ m^2^ plant^–1^ in Heidou (*P* < 0.001, Figure 3D).

There was a large variation among the 43 genotypes in root physiological traits including the total amount of carboxylates and acid phosphatase activity (*P* < 0.001 for both) (Figures 3E,F). We found a 35-fold difference in the activity of acid phosphatase in the rhizosheath among 43 genotypes, ranging from 0.19 nkat g^–1^ soil DW in Baiqidawandou to 6.63 nkat g^–1^ soil DW in Xinyangyangyandou (*P* < 0.001, Figure 3E). Similarly, the amount of carboxylates in the rhizosheath soil relative to root dry weight also differed seven-fold among genotypes, ranging from 14.9 μmol g^–1^ root DW in Pixianlayanghuang to 110.8 μmol g^–1^ root DW in Datunxiaoheidou (*P* < 0.001, Figure 3F). The composition of carboxylates showed a large variation among 43 genotypes (*P* < 0.001, Figure 3G). All 43 genotypes showed a predominant combination of malonate and malate in the rhizosheath soil, whereas other carboxylates including fumarate and *trans*-aconitate only accounted for 0.25–67.5% of total carboxylates (Supplementary Figure 2). Malonate accounted for the largest proportion of the total carboxylates in the rhizosheath soil (19–85%, *P* < 0.001) (Supplementary Figure 2). There was a large variation in the proportion of malate (10–43%, *P* < 0.001) and succinate (0–33%, *P* < 0.001, Supplementary Figure 2).

### Correlations Among Traits

**FIGURE 4:**
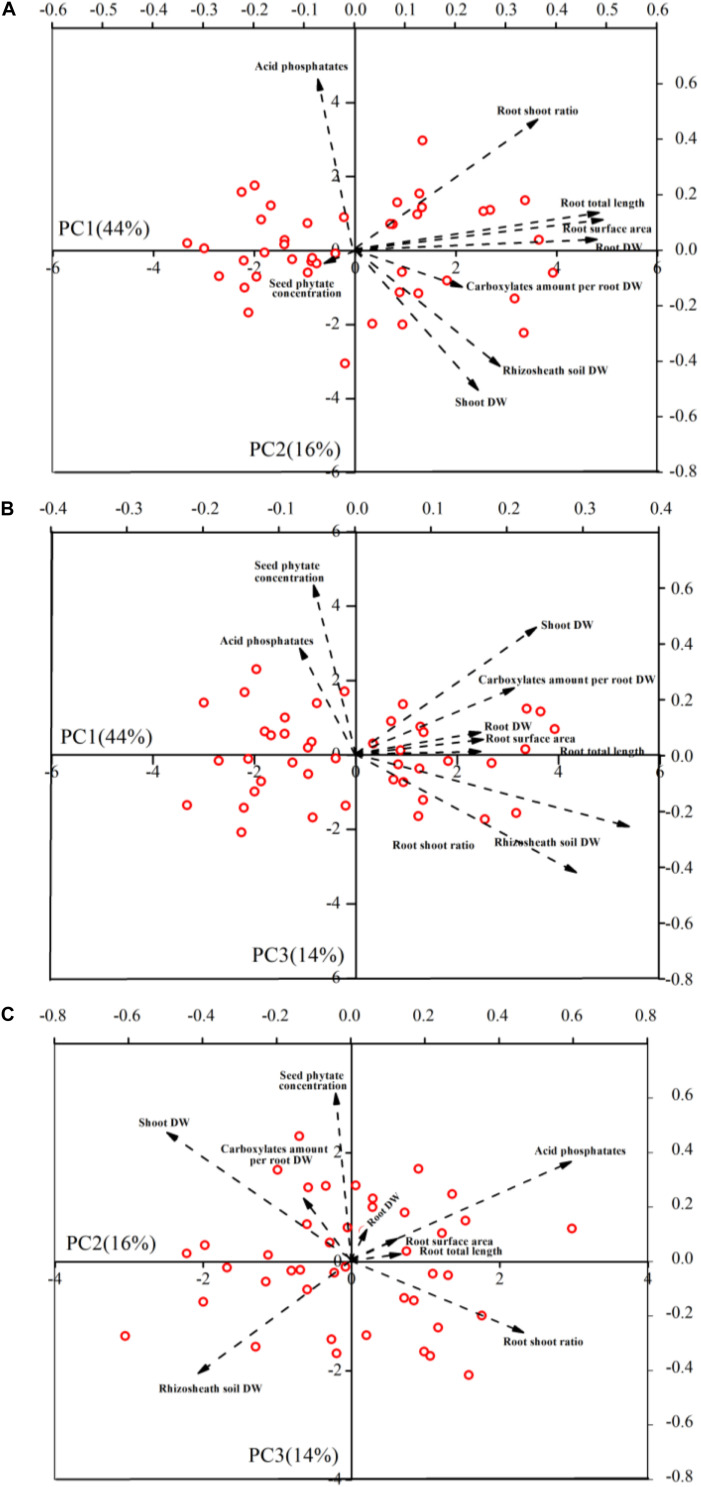
Principal component analyses of 9 plant traits for 43 soybean genotypes. Biplot vectors are trait factor loadings, whereas the position of each genotype is shown. Panels refers to PC1 vs. PC2 **(A)**, PC1 vs. PC3 **(B)**, and PC2 vs. PC3 **(C)**, respectively.

Based on nine plant traits of 43 genotypes, PCA explained 74% of the variation in the first, second, and third components (Figures 4A,B and Supplementary Table 3). The first component (PC1) represented 44% of the variance and primarily comprised root DW, total root length and root surface area. The second component represented 16% of variability and accounted mainly for shoot DW, root: shoot ratio, acid phosphatase activity and rhizosheath soil DW. The third component (PC3) represented 14% of variability, and primarily accounted for seed phytate-P concentration, acid phosphatase activity and shoot DW (Figures 4A–C and Supplementary Table 3).

Seed phytate-P concentrations showed no significant correlation with either root size, root morphological traits, physiological traits, or shoot DW (Table 1). Root DW had a significant positive correlation with all plant traits, except acid phosphatase activity, whereas acid phosphatase activity was only significantly correlated with rhizosheath soil DW. Total root length, root surface area and the total amount of carboxylates showed a significant positive correlation with all other traits, except seed phytate-P concentration and acid phosphatase activity (Table 1).

**TABLE 1:** Pearson’s correlation matrix for 9 plant traits in 43 soybean genotypes.

	Phytate-P	Root DW	Shoot DW	RhiS DW	Root/shoot ratio	TRL	RSA	APase
Root DW	−0.04							
Shoot DW	0.08	0.54***						
RhiS DW	−0.25	0.40**	0.36**					
Root/shoot ratio	−0.11	0.71***	−0.21	0.16				
TRL	−0.05	0.83***	0.34*	0.47**	0.68***			
RSA	−0.04	0.90***	0.41**	0.43**	0.70***	0.97***		
APase	0.10	−0.12	−0.11	−0.38**	−0.06	−0.06	−0.06	
Carb_root	−0.10	0.31*	0.28	0.21	0.11	0.30	0.33*	−0.01

Root DW, root dry weight; Shoot DW, shoot dry weight; RhiS DW, rhizosheath soil DW; TRL, total root length; RSA, root surface area; APase, acid phosphatase activity in the rhizosheath soil; Carb_root, the amount of total carboxylates relative to root dry weight. Significant correlations are shown in bold,* *P* < 0.05;**P < 0.01; ***P < 0.001.

### Classification of Soybean Genotypes

There were 19 (out of 43) soybean genotypes that had a lower seed phytate-P concentration and at least one desirable P-acquisition trait (Table 2). The soybean genotypes showed a large variation with respect to each trait (Supplementary Figure 3 and Table 2). The maximum and minimum comprehensive score was gained by genotype Diliuhuangdou-2, Siliyuan and Jidou no 7 (5 out of 6), and genotype Lvcaodou, Zheng84240-B1, Miyangxiaozihuang, Binhaidahuangkezijia, Baiqidawandou and Zheng8516 (1 out of 6), respectively. Three soybean genotypes showed a higher score (5 out of 6)’ of these, two genotypes had seed phytate-P concentration below 1.53 mg g^–1^ DW including Siliyuan and Diliuhuangdou-2. The high-score genotypes all exhibited efficient root morphological traits (Table 2).

**TABLE 2:** Comprehensive plant performance scores of low phytate-P soybean genotypes.

Genotype name	Phytate-P Conc mg g^–1^	Root size		Root morphological trait		Root physiological trait	
		Root DW	RhiS DW	TRL	RSA	APase	Carb_root
Lvcaodou	0.69					√	
**Siliyuan**	0.95	√	√	√	√		√
Pingdinghei	1.01		√	√	√	√	
Huaheihu	1.07	√		√	√		
Gaozuoxuan no. 1	1.16	√	√	√	√		
**Diliuhuangdou-2**	1.53	√	√	√	√	√	
Pixiannianzhuangliuyuexian	2.09		√			√	√
Zhechengxiaohuangdou	2.11					√	√
Jidou no. 7	2.27	√	√	√	√		√
Chadou	2.28	√		√	√		
Bo’aihongpizaojiaozi	2.48					√	√
Tongshanqingdadou	2.49	√	√		√		√
Miyangxiaozihuang	2.50					√	
Zheng8516	2.55		√				
Pixiansilicao	2.56	√		√	√	√	
Yangtianxioahuangdou	2.58		√	√	√		√
Binhaidahuangkezijia	2.77					√	
Miyangniumaohuang	2.79	√	√				√
Baiqidawandou	2.87		√				

*Root DW, root dry weight; RhiS DW, rhizosheath soil dry weight; TRL, total root length; RSA, root surface area; APase, acid phosphate activity in the rhizosheath soil; Carb_root, the total amount of total carboxylates relative to root DW. Ticks mean that genotypes have lower seed phytate-P concentration than the mean seed phytate-P concentration of 43 soybean genotypes, and higher amount or larger root traits than the average value. Genotype with bold is the recommended genotypes.*

## Discussion

A vital first step for improving trait in breeding programs is to explore the genetic variation in germplasm (Perera et al., 2019). The soybean core collections with different sample size were established based on the genetic diversity existed among its originated country of China (Wang et al., 2006; Qiu et al., 2013), in which the mini core collection had the rich diversity with the least sample size (Song et al., 2010). Thus, they had priority for discovering new traits or genes. The present study shows a large variation in seed phytate-P concentration among 256 soybean genotypes (Figure 1) because almost all mini core collection was included. Since the present genotypes were grown at the same time in the same field, we minimized environmental variables, growing location, irrigation condition, fertilizer applications soil type, and planting time (Boehm et al., 2017). Therefore, variation in seed phytate-P concentration in the present study can be primarily attributed to the broad genotypic variability of 256 soybean genotypes. This agrees with results of Raboy et al. (1984), who found extensive variation in soybean seed phytate-P concentration among 38 genotypes that were grown at the same time in the same field. Seed phytate P concentration ranged from 13.9 to 23.0 mg g^–1^ DW with a mean of 17.6 mg g^–1^ DW. Likewise, Horner et al. (2005) found a large variation in phytate-P concentration among 86 soybean genotypes that were grown at the same time at three locations, ranging from 7.7 to 22.2 mg g^–1^ DW with a mean of 14.5 mg g^–1^ DW. These results provide compelling evidence that genotypic variation in phytate-P concentration exists within soybean germplasm, despite a previous study showing that soybean has higher concentrations of seed phytate-P compared with our study (Wilcox et al., 2000).

Importantly, we found that more than half of the studied genotypes had a lower seed phytate P concentration than that of Gm-lpa-ZC-2 (range from 5.6 to 9.7 mg g^–1^ DW) and Gm-lpa-TW75-1 (range from 3.0 to 6.4 mg g^–1^ DW), although both Gm-lpa-ZC-2 and Gm-lpa-TW75-1 were previously identified as low-phytate soybean mutants (Yuan et al., 2007, 2009). This offers the exciting possibility of identifying genotypes with even lower phytate-P concentrations than known before. This information is highly valuable for breeding and identifying soybean genotypes with low phytate-P concentrations, because seed phytate-P not only reduces the availability of micro-nutrients, especially Zn, Fe, and Cu, for humans and livestock, contributing to malnutrition, but also cannot be efficiently utilized by humans and non-ruminant animals, contributing to high losses of P to the environment (Raboy, 2001).

As we hypothesized, we found that under low-P conditions, root size (root dry weight), rhizosheath soil dry weight, morphological (total root length and root surface area) and physiological (carboxylate exudation and acid phosphate activity in rhizosheath soil) traits showed significant variations among 43 soybean genotypes (Figure 3), confirming a large genotypic variability for these traits. Root traits are critically important by determining soil exploration and therefore nutrient acquisition (Lynch, 2007). They affect a plant’s acquisition of P including through the release of root exudates such as carboxylates and enzymes that alter the rhizosheath soil properties and increase the amount of available nutrients, distribution of roots, and morphological characteristic of the root system such as root surface area and specific root length. All of these traits influence the soil volume that is explored by the root system for acquisition of nutrients (Lambers et al., 2006; Richardson et al., 2011; White et al., 2013; Krishnapriya and Pandey, 2016; Zhou et al., 2016; Lynch, 2019; Wang et al., 2019; White, 2019). Therefore, this information provides a scientific basis for breeding and identifying soybean genotypes with greater P-acquisition efficiency.

The absence of correlations between desirable root traits and seed phytate-P concentration gives valuable insight for selection in breeding programs. In this study, principal component analysis explained 74% of variation in the first, second, and third components (Figures 4A–C and Supplementary Table 3). The first (PC1) and second (PC2) components primarily comprised all of the root traits measured, whereas the third component (PC3) comprised seed phytate-P concentration. Our study shows that seed phytate-P concentration had no significant correlation with any root trait measured, while there was a significant correlation among all of these root traits measured (Table 1 and Supplementary Figure 3). This suggests that there is potential to breed low seed phytate-P soybean genotypes without compromising desirable P-acquisition traits. Classifying the genotypes based on the seed phytate-P concentration and root traits is a useful tool in selecting potential genotypes to be used in biofortification of well-adapted genotypes with enhanced P acquisition. In the present study, we identified 19 soybean genotypes belonging to a low-phytate group with desirable root traits based on the plant performance score (Table 2 and Supplementary Figure 3), while some genotypes only had a low score (1 out of 6). The low-phytate soybean genotypes with desirable root traits identified in the present study are recommended to be used as genetic resource for breeding programs, whereas the low-phytate soybean genotypes with undesirable traits could be used as experimental materials in future genomic studies on low seed phytate. Although there were three genotypes that had a higher score (5 out of 6), we selected two soybean genotypes including Siliyuan and Diliuhuangdou-2 with both highly desirable P-acquisition traits and low phytate-P concentrations (Table 2), because these had far lower seed phytate-P concentration than the other two genotypes. In addition, seed phytate-P concentrations of these genotypes were lower than that of Gm-lpa-ZC-2 and Gm-lpa-TW75-1, which were previously identified as low phytate-P soybean mutants (Yuan et al., 2007, 2009), suggesting the possibility of identifying and selecting further low-phytate soybean genotypes with highly desirable P-acquisition traits. Importantly, these two genotypes have crop yields of more than 3 t ha^–1^ (unpublished), holding promise for application in agriculture. Further work on agronomic and physiological traits as well as seed quality parameters of the low-phytate P genotypes is needed. For example, it is important to examine whether seed P content of the low-phytate P genotype is sufficient for seed establishment.

In summary, selecting low phytate-P soybean genotypes with desirable P-acquisition traits will tighten the P cycle in crop production systems, animal production systems as well as human consumption, and lead to reduction of P-fertilizer cost, increase of nutritional quality of soymeal, and decrease of P-related environmental pollution.

## Conclusion

We show substantial genotypic variation in seed phytate-P concentration and a range of root traits related to P acquisition. Some of the present genotypes showed even lower seed phytate-P concentrations than mutants that were considered the best available in terms of low seed phytate-P. Most importantly, seed phytate-P concentration was not correlated with any desirable root traits measured in this study.

## Data Availability Statement

The generated datasets available by request to the corresponding author.

## Author Contributions

MK, HL, JP, and W-FC designed the study. MK and WJ performed the experiments and collected the data. MK analyzed the data. MK, HL, JJ, L-JQ, JP, WD, W-FC, and FZ interpreted the data and wrote the manuscript. All authors contributed to the article and approved the submitted version.

## Conflict of Interest

The authors declare that the research was conducted in the absence of any commercial or financial relationships that could be construed as a potential conflict of interest.

## Supplementary Material

Supplementary Figure 1 Spatial distribution characteristics of phytate phosphorus (P) concentrations of 256 soybean genotypes. Abbreviations used along the *x*-axis refer to the provinces, spelled out toward the right of the figure panel.

Supplementary Figure 2 The proportion of carboxylates in the rhizosheath soil consisting of malate, citrate, fumarate, succinate, and *trans*-aconitate of 43 soybean genotypes.

Supplementary Figure 3 Plant performance scores of 43 soybean genotypes grown in washed river sand with a low supply of phosphorus (P).

Supplementary Table 1 Detailed information on the 256 soybean genotypes used in the present study to measure seed phytate phosphorus concentration, and to study six root traits, associated with phosphorus acquisition.

Supplementary Table 2 Descriptive statistics of phytate-phosphorus (P) concentration of 256 soybean genotypes commonly cultivated in China.

Supplementary Table 3 Variable scores of nine plants traits in 43 soybean genotypes and proportion of variation of each component.

## References

[B1] Alvey S. Bagayoko M. Neumann G. Buerkert A. (2001). Cereal/legume rotations affect chemical properties and biological activities in two West African soils. *Plant Soil* 231 45–54. 10.1023/A:1010386800937

[B2] Ao J. Fu J. Tian J. Yan X. Liao H. (2010). Genetic variability for root morph-architecture traits and root growth dynamics as related to phosphorus efficiency in soybean. *Funct. Plant Biol.* 37 304–312. 10.1071/FP09215

[B3] Bilal H. M. Aziz T. Maqsood M. A. Farooq M. Yan G. J. (2018). Categorization of wheat genotypes for phosphorus efficiency. *PLoS One* 13:e0205471. 10.1371/journal.pone.0205471 30332479 PMC6192622

[B4] Boehm J. D. Walker F. R. Bhandari H. S. Kopsell D. Pantalone V. R. (2017). Seed inorganic phosphorus stability and agronomic performance of two low-phytate soybean lines evaluated across six southeastern US environments. *Crop Sci.* 57 2555–2563. 10.2135/cropsci2017.02.0107

[B5] Brinch P. H. Sorensen L. D. Holm P. B. (2002). Engineering crop plants: getting a handle on phosphate. *Trend Plant Sci.* 7 118–125. 10.1016/S1360-1385(01)02222-1 11906835

[B6] Cong W.-F. Hoffland E. Li L. Six J. Sun J. Bao X. (2015). Intercropping enhances soil carbon and nitrogen. *Global Change Biol.* 21 1715–1726. 10.1111/gcb.12738 25216023

[B7] Cong W.-F. Suriyagoda L. Lambers H. (2020). Tightening the phosphorus cycle through phosphorus-efficient crop genotypes. *Trend Plant Sci.* 10 967–975. 10.1016/j.tplants.2020.04.013 32414603

[B8] Cordell D. Drangert J. O. White S. (2009). The story of phosphorus: global food security and food for thought. *Global Environ. Change* 19 292–305. 10.1016/j.gloenvcha.2008.10.009

[B9] Dinkelaker B. Römheld V. Marschner H. (1989). Citric-acid excretion and precipitation of calcium citrate in the rhizosphere of White Lupin (*Lupinus abus L*). *Plant Cell Environ.* 12 285–292. 10.1111/j.1365-3040.1989.tb01942.x

[B10] Fernandez M. C. Rubio G. (2015). Root morphological traits related to phosphorus-uptake efficiency of soybean, sunflower, and maize. *J. Plant Nutr. Soil Sci.* 178 807–815. 10.1002/jpln.201500155

[B11] Gao X. Wu M. Xu R. Wang X. Pan R. Kim H. (2014). Root interactions in a maize/soybean intercropping system control soybean soil-borne disease, red crown rot. *PLoS One* 9:e95031. 10.1371/journal.pone.0095031 24810161 PMC4014482

[B12] Ghaffar S. Stevenson R. J. Khan Z. (2017). Effect of phosphorus stress on Microcystis aeruginosa growth and phosphorus uptake. *PLoS One* 12:e0174349. 10.1371/journal.pone.0174349 28328927 PMC5362216

[B13] Hinsinger P. (2001). Bioavailability of soil inorganic P in the rhizosphere as affected by root-induced chemical changes: a review. *Plant Soil* 237 173–195. 10.1023/A:1013351617532

[B14] Holford I. C. R. (1997). Soil phosphorus: its measurement, and its uptake by plants. *Aus. J. Soil Res.* 35 227–239. 10.1071/S96047

[B15] Horner H. T. Cervantes M. T. Healy R. Reddy M. B. Deardorff B. L. Bailey T. B. (2005). Oxalate and phytate concentrations in seeds of soybean genotypes [*Glycine max (L.) Merr.]*. *J. Agric. Food Chem.* 53 7870–7877. 10.1021/jf051193i 16190644

[B16] Krishnapriya V. Pandey R. (2016). Root exudation index: screening organic acid exudation and phosphorus acquisition efficiency in soybean genotypes. *Crop Past. Sci.* 67 1096–1109. 10.1071/CP15329

[B17] Lambers H. Shane M. W. Cramer M. D. Pearse S. J. Veneklaas E. J. (2006). Root structure and functioning for efficient acquisition of phosphorus: matching morphological and physiological traits. *Ann. Bot.* 98 693–713. 10.1093/aob/mcl114 16769731 PMC2806175

[B18] Liang Q. Cheng X. Mei M. Yan X. Liao H. (2010). QTL analysis of root traits as related to phosphorus efficiency in soybean. *Ann. Bot.* 106 223–234. 10.1093/aob/mcq097 20472699 PMC2889805

[B19] Lynch J. P. (2007). Roots of the second green revolution. *Aus. J. Bot.* 55 493–512. 10.1071/BT06118

[B20] Lynch J. P. (2019). Root phenotypes for improved nutrient capture: an underexploited opportunity for global agriculture. *New Phytol.* 223 548–564. 10.1111/nph.15738 30746704

[B21] Lyu Y. Tang H. L. Li H. Zhang F. Rengel Z. Whalley W. R. (2016). Major crop species show differential balance between root morphological and physiological responses to variable phosphorus supply. *Front. Plant Sci.* 7:1939. 10.3389/fpls.2016.01939 28066491 PMC5174099

[B22] Maharjan P. Penny J. Partington D. L. Panozzo J. F. (2019). Genotype and environment effects on the chemical composition and rheological properties of field peas. *J. Sci. Food Agric.* 99 5409–5416. 10.1002/jsfa.9801 31077380

[B23] Marschner H. (1998). Role of root growth, arbuscular mycorrhiza, and root exudates for the efficiency in nutrient acquisition. *Field Crops Res.* 56 203–207. 10.1016/S0378-4290(97)00131-7

[B24] Nuruzzaman M. Lambers H. Bolland M. D. A. Veneklaas E. J. (2006). Distribution of carboxylates and acid phosphatase and depletion of different phosphorus fractions in the rhizosphere of a cereal and three grain legumes. *Plant Soil* 281 109–120. 10.1007/s11104-005-3936-2

[B25] Pang J. Ryan M. H. Siddique K. H. M. Simpson R. J. (2017). Unwrapping the rhizosheath. *Plant Soil* 418 129–139. 10.1007/s11104-017-3358-y

[B26] Pang J. Zhao H. Bansal R. Bohuon E. Lambers H. Ryan M. H. (2018). Leaf transpiration plays a role in phosphorus acquisition among a large set of chickpea genotypes. *Plant Cell Environ.* 41 2069–2079. 10.1111/pce.13139 29315636

[B27] Pearse S. J. Veneklaas E. J. Cawthray G. Bolland M. D. A. Lambers H. (2007). Carboxylate composition of root exudates does not relate consistently to a crop species’ ability to use phosphorus from aluminium, iron or calcium phosphate sources. *New Phytol.* 173 181–190. 10.1111/j.1469-8137.2006.01897.x 17176404

[B28] Perera I. Fukushima A. Arai M. Yamada K. Nagasaka S. Seneweera S. (2019). Identification of low phytic acid and high Zn bioavailable rice (*Oryza sativa* L*.) from* 69 accessions of the world rice core collection. *J. Cereal Sci.* 85 206–213. 10.1016/j.jcs.2018.12.010

[B29] Qiu L.-J. Xing L. Guo Y. Wang J. Jackson S. A. Chang R. (2013). A platform for soybean molecular breeding: the utilization of core collections for food security. *Plant Mol. Biol.* 83 41–50. 10.1008/S11103-013-0076-6 23708950 PMC3755216

[B30] Raboy V. (2001). Seeds for a better future: ‘low phytate’, grains help to overcome malnutrition and reduce pollution. *Trends Plant Sci.* 6 458–462. 10.1016/S1360-1385(01)02104-5 11590064

[B31] Raboy V. Dickinson D. B. Below F. E. (1984). Variation in seed total phosphorus, phytic acid, zinc, calcium, magnesium, and protein among lines of *Glycine max* and *Glycine soja*. *Crop Sci.* 24 431–434. 10.2135/cropsci1984.0011183X002400030001x

[B32] Raghothama K. G. Karthikeyan A. S. (2005). Phosphate acquisition. *Plant Soil* 274 37–49. 10.1007/s11104-004-2005-6

[B33] Richardson A. E. Lynch J. P. Ryan P. R. Delhaize E. Smith F. A. Smith S. E. (2011). Plant and microbial strategies to improve the phosphorus efficiency of agriculture. *Plant Soil* 349 121–156. 10.1007/s11104-011-0950-4

[B34] Salvagiotti F. Cassman K. G. Specht J. E. Walters D. T. Weiss A. Dobermann A. (2008). Nitrogen uptake, fixation and response to fertilizer N in soybeans: a review. *Field Crops Res.* 108 1–13. 10.1016/j.fcr.2008.03.001

[B35] Sharpley A. Foy B. Withers P. (2000). Practical and innovative measures for the control of agricultural phosphorus losses to water: an overview. *J. Environ. Qual.* 29 1–9. 10.2134/jeq2000.00472425002900010001x

[B36] Shen J. Rengel Z. Tang C. Zhang F. (2003). Role of phosphorus nutrition in development of cluster roots and release of carboxylates in soil-grown *Lupinus albus*. *Plant Soil* 248 199–206. 10.1023/A:1022375229625

[B37] Song X. Li Y. Chang R. Guo P. Qiu L.-J. (2010). Population structure and genetic diversity of mini core collection of cultivated soybean (*Glycine max*) in China. *Sci. Agric. Sin.* 43 2209–2219. 10.3864/j.issn.0578-1752.2010.11.00

[B38] Wang L. Guan R. Li Y. Lin F. Qiu L.-J. (2007). Genetic diversity of Chinese spring soybean germplasm revealed by SSR markers. *Plant Breed.* 127 56–61. 10.1111/j.1439-0523.2007.01429.x

[B39] Wang L. Guan Y. Guan R. Li Y. Ma Y. Dong Z. (2006). Establishment of Chinese soybean (*Glycine max*) core collection with agronomic traits and SSR markers. *Euphytica* 151 215–223. 10.1007/s10681-006-9142-3

[B40] Wang W. Ding G. White P. J. Wang X. Jin K. Xu F. (2019). Mapping and cloning of quantitative trait loci for phosphorus efficiency in crops: opportunities and challenges. *Plant Soil* 439 91–112. 10.1007/s11104-018-3706-6

[B41] White P. J. (2019). Root traits benefitting crop production in environments with limited water and nutrient availability. *Ann. Bot.* 124 883–890. 10.1093/aob/mcz162 31599920 PMC6881216

[B42] White P. J. George T. S. Dupuy L. X. Karley A. J. Valentine T. (2013). Root traits for infertile soils. *Front. Plant Sci.* 4:193. 10.3389/fpls.2013.00193 23781228 PMC3678079

[B43] Wilcox J. R. Premachandra G. S. Young K. A. Raboy V. (2000). Isolation of high seed inorganic P, low-phytate soybean mutants. *Crop Sci.* 40 1601–1605. 10.2135/cropsci2000.4061601x

[B44] Xia H. Wang Z. Zhao J. Sun J. Bao X. Christie P. (2013). Contribution of interspecific interactions and phosphorus application to sustainable and productive intercropping systems. *Field Crops Res.* 154 53–64. 10.1016/j.fcr.2013.07.011

[B45] Yuan F. Zhao H. Ren X. Zhu S. Fu X. Shu Q. (2007). Generation and characterization of two novel low phytate mutations in soybean (*Glycine max* L. Merr.). *Theor. Appl. Genet.* 115 945–957. 10.1007/s00122-007-0621-2 17701395

[B46] Yuan F. Zhu D. Deng B. Fu X. Dong D. Zhu S. (2009). Effects of two low phytic acid mutations on seed quality and nutritional traits in soybean (*Glycine max L*. Merr). *J. Agric. Food Chem.* 57 3632–3638. 10.1021/jf803862a 19323582

[B47] Zhou T. Du Y. Ahmed S. Liu T. Ren M. Liu W. (2016). Genotypic differences in phosphorus efficiency and the performance of physiological characteristics in response to low phosphorus stress of soybean in southwest of China. *Front. Plant Sci.* 7:1776. 10.3389/fpls.2016.01776 27933086 PMC5121124

