# Supplementary material for: Targeting Low-Phytate Soybean Genotypes Without Compromising Desirable Phosphorus-Acquisition Traits
Source: Front Genet. 2020 Dec 14;11:574547. doi: 10.3389/fgene.2020.574547 (PMC7767974; doi:10.3389/fgene.2020.574547)
Supplement: Supplementary file 1 [file Data_Sheet_1.PDF]

Supplementary Material

Breeding low-phytate soybean genotypes without compromising desirable phosphorus-acquisition traits

Mireadili Kuerban, WenFeng Jiao, Jiayin Pang, Jingying Jing, Li-Juan Qiu, Wenli Ding, Wen-Feng Cong, Fusuo Zhang and Hans Lambers

1 Supplementary Figures and Tables

1.1 Supplementary Figures

FIGURE S1| Spatial distribution characteristics of phytate phosphorus (P) concentrations of 256 soybean genotypes. Abbreviations used along the x-axis refer to the provinces, spelled out towards the right of the figure panel.

FIGURE S2| The proportion of carboxylates in the rhizosheath soil consisting of malate, citrate, fumarate, succinate and *trans*-aconitate of 43 soybean genotypes.

FIGURE S3| Plant performance scores of 43 soybean genotypes grown in washed river sand with a low supply of phosphorus (P).

1.2 Supplementary Tables

TABLE S1| Detailed information on the 256 soybean genotypes used in the present study to measure seed phytate phosphorus concentration,and to study six root traits, associated with phosphorus acquisition.

TABLE S2| Descriptive statistics of phytate-phosphorus (P) concentration of 256 soybean genotypes commonly cultivated in China.

TABLE S3| Variable scores of nine plants traits in 43 soybean genotypes and proportion of variation of each component.

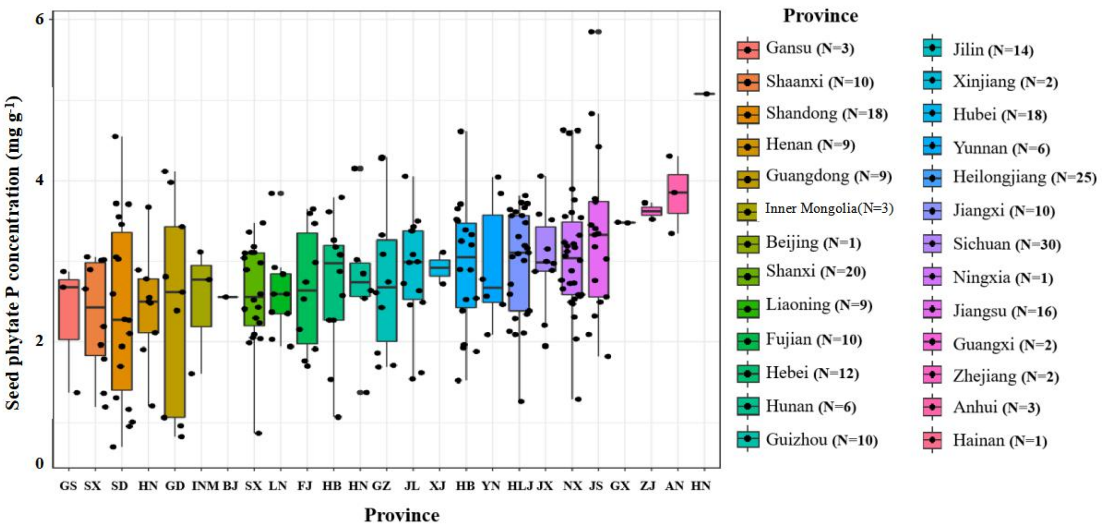

FIGURE S1| Spatial distribution characteristics of phytate phosphorus (P) concentrations of 256 soybean genotypes. Abbreviations used along the x-axis refer to the provinces, spelled out towards the right of the figure panel.

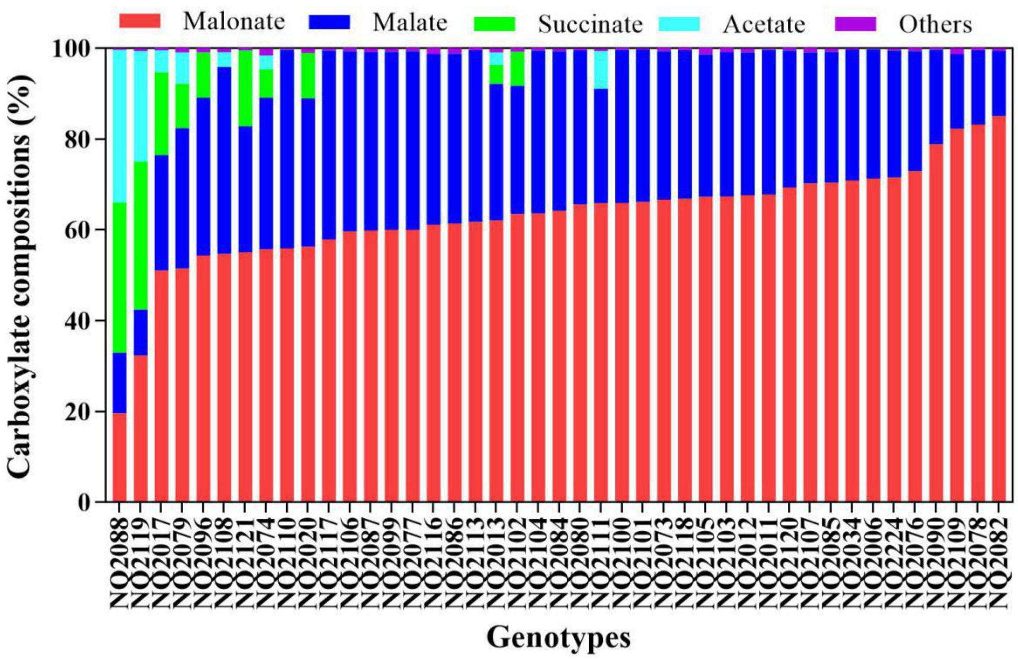

FIGURE S2| The proportion of carboxylates in the rhizosheath soil consisting of malonate, malate, succinate and acetate and other acids of 43 soybean genotypes.

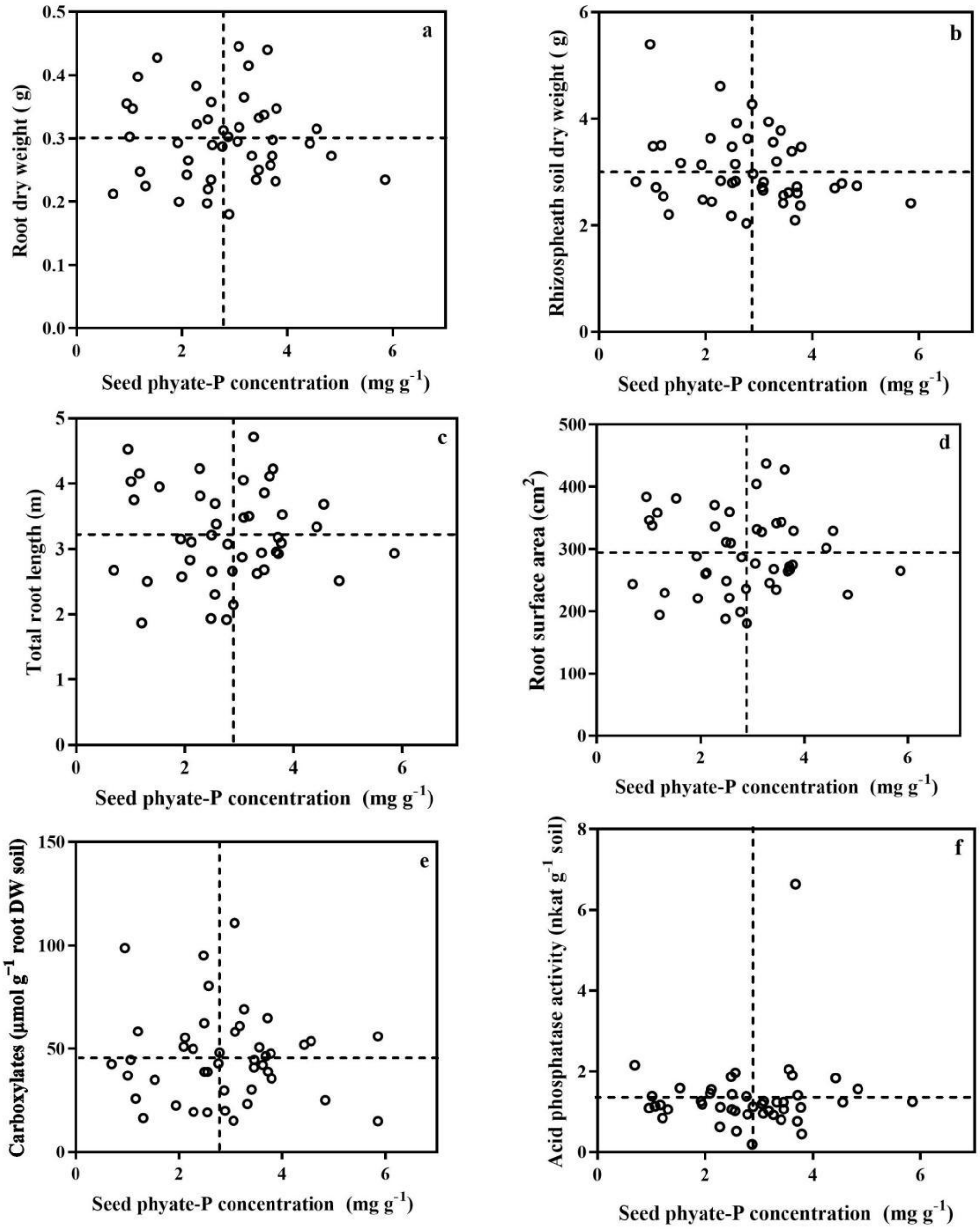

FIGURE S3| Plant performance scores of 43 soybean genotypes grown in washed river sand with a low supply of phosphorus (P). Data are the mean value of four replicates.

TABLE S1| Detailed information on the 256 soybean genotypes used in the present study to measure seed phytate phosphorus concentration,and to study six root traits, associated with phosphorus acquisition. Y represent those 43 soybean genotypes were included for the pot experiment.

Genotype code	Genotype name	Type	Origin	Region	Included root traits
NQ0002		phyA2	Eastern Europe		
NQ0003		phyA2	Eastern Europe		
NQ0016		phyA2	Russia		
NQ0258		phyA2	Russia		
NQ0286	Mohemoshidou	phyA2	Heilongjiang	Northeast China plain	
NQ0333	Heijian no1	phyA2	Heilongjiang	Northeast China plain	
NQ0350	Panshidou	phyA2	Jilin	Northeast China plain	
NQ0366	Heihe101	phyA2	Heilongjiang	Northeast China plain	
NQ0382	Heihe13	phyA2	Heilongjiang	Northeast China plain	
NQ0409	Heihe40	phyA2	Heilongjiang	Northeast China plain	
NQ0410	Heihe41	phyA2	Heilongjiang	Northeast China plain	
NQ0436	Heihe49	phyA2	Heilongjiang	Northeast China plain	
NQ0437	Heihe44	phyA2	Heilongjiang	Northeast China plain	
NQ0508	Heihe13	phyA2	Heilongjiang	Northeast China plain	
NQ0576	Daqingdou	RINO1	Shanxi	Loess Plateua	
NQ0683	Heihe no1	phyA2	Heilongjiang	Northeast China plain	
NQ0785	Pingdinghuang	PSTOL1	Shandong	Huang-Huai-Hai plain	

NQ0786	Qiyueluan	PSTOL1	Shandong	Huang-Huai-Hai plain
NQ0812	Ninglingtian'edan	RINO1	Henan	Huang-Huai-Hai plain
NQ0934	Shengli no3	phyA2	Shandong	Huang-Huai-Hai plain
NQ0938	Dahuangdou	PSTOL1	Shandong	Huang-Huai-Hai plain
NQ0953	Pingdinghuang	PSTOL1	Shandong	Huang-Huai-Hai plain
NQ1005	Hongpihuangdou	PSTOL1	Hubei	Middle-Lower Yangtze plain
NQ1007	Tianbadou	PSTOL1	Siquan	Sichuan Basin
NQ1019	Pengxiliuyuehuang	PSTOL1	Siquan	Sichuan Basin
NQ1097	Bayuezao-2	PSTOL1	Guizhou	Yunnan-Guizhou plateau
NQ1145	Songziyanghuangdou	PSTOL1	Hubei	Middle-Lower Yangtze plain
NQ1198	Dahuadou	PSTOL1	Siquan	Sichuan Basin
NQ1205	Chongqingjiuyuehuang	PSTOL1	Siquan	Sichuan Basin
NQ1300	Xiaoheidou	phyA2	Yunnan	Yunnan-Guizhou plateau
NQ1310	Changlingqingdou	PSTOL1	Anhui	Middle-Lower Yangtze plain
NQ1393	Pudou451	PSTOL1	Fujian	Middle-Lower Yangtze plain
NQ1444	Jim	phyA2	America	
NQ1626	Yushulinzihuangdou	phyA2	Hebei	Huang-Huai-Hai plain
NQ1738	Mengdou no32	phyA2	Inner-Mongolia	Northen arid and semiarid region
NQ1740	Hu1414	phyA2	Inner-Mongolia	Northen arid and semiarid region
NQ1790	Hefeng37	phyA2	Heilongjiang	Northeast China plain
NQ2001	Xiaoheidou	Core collection	Sha'anxi	Loess Plateua
NQ2002	Youhuangdou	Core collection	Gansu	Northen arid and semiarid region
NQ2003	Tianedan	Core collection	Shanxi	Loess Plateua
NQ2004	Dongshan69	Core collection	Shanxi	Loess Plateua
NQ2005	Lvhuangdou	Core collection	Gansu	Northen arid and semiarid region

NQ2006	Baiqidawandou	Core collection	Hebei	Huang-Huai-Hai plain	
NQ2007	Xiaohuangdou	Core collection	Shanxi	Loess Plateua	
NQ2008	Daheidou	Core collection	Shanxi	Loess Plateua	
NQ2009	Xiaheidou	Core collection	Shanxi	Loess Plateua	
NQ2010	Yanqihuangdou	Core collection	Xinjiang	Northen arid and semiarid region	
NQ2011	Yangtianxioahuangdou	Core collection	Hebei	Huang-Huai-Hai plain	Y
NQ2012	Chichenglvhuangdou	Core collection	Hebei	Huang-Huai-Hai plain	Y
NQ2013	Dadunxiaoheidou	Core collection	Hebei	Huang-Huai-Hai plain	Y
NQ2014	Huangdou2	Core collection	Shanxi	Loess Plateua	
NQ2015	Xiaohuangdou	Core collection	Shanxi	Loess Plateua	
NQ2016	Liushiribaidou	Core collection	Shanxi	Loess Plateua	
NQ2017	Xiataizimoshidou	Core collection	Hebei	Huang-Huai-Hai plain	Y
NQ2018	Lvpihuangdou	Core collection	Shanxi	Loess Plateua	
NQ2019	Baipihuangdou	Core collection	Shanxi	Loess Plateua	
NQ2020	Miyunlaoyelian	Core collection	Beijing	Huang-Huai-Hai plain	Y
NQ2021	Nidinghuameidou	Core collection	Ningxia	Northen arid and semiarid region	
NQ2022	Tian'e'dan	Core collection	Shanxi	Loess Plateua	
NQ2023	Huanggandou	Core collection	Shanxi	Loess Plateua	
NQ2024	Changjihuangdou	Core collection	Xinjiang	Northen arid and semiarid region	
NQ2025	Xiaohuangdou	Core collection	Shanxi	Loess Plateua	
NQ2026	Yuxuan no13	Core collection	Shanxi	Loess Plateua	
NQ2027	Bailudou	Core collection	Shanxi	Loess Plateua	
NQ2028	Zaoshuhungdou	Core collection	Sha'anxi	Loess Plateua	
NQ2029	Xiaobaidou2	Core collection	Shanxi	Loess Plateua	
NQ2030	Huipizhiheidou	Core collection	Shanxi	Loess Plateua	

NQ2031	Xiaoyuanhuangdou	Core collection	Shanxi	Loess Plateua	
NQ2032	Xiaoheidou	Core collection	Sha'anxi	Loess Plateua	
NQ2033	Tu'eryan	Core collection	Hebei	Huang-Huai-Hai plain	Y
NQ2034	Nan guanxiaopiqing	Core collection	Hebei	Huang-Huai-Hai plain	Y
NQ2035	Laoheidou	Core collection	Sha'anxi	Loess Plateua	
NQ2036	Heinong no2	Core collection	Heilongjiang	Northeast China plain	
NQ2037	Kebei no1	Core collection	Heilongjiang	Northeast China plain	
NQ2038	Changchunmancangjin	Core collection	Jilin	Northeast China plain	
NQ2039	Daliheidou	Core collection	Liaoning	Northeast China plain	
NQ2040	Beidebaihuadali	Core collection	Jilin	Northeast China plain	
NQ2041	Jinshanchamoshidou	Core collection	Jilin	Northeast China plain	
NQ2042	Jilinchalihua	Core collection	Jilin	Northeast China plain	
NQ2043	Heimoshidou	Core collection	Jilin	Northeast China plain	
NQ2044	Jinzhou4-1	Core collection	Liaoning	Northeast China plain	
NQ2045	Tonghuapingdingxiang	Core collection	Jilin	Northeast China plain	
NQ2046	Mufeng no1	Core collection	Heilongjiang	Northeast China plain	
NQ2047	Suinong no1	Core collection	Heilongjiang	Northeast China plain	
NQ2048	Qinganheidou	Core collection	Heilongjiang	Northeast China plain	
NQ2049	Fangzhengmoshidou	Core collection	Heilongjiang	Northeast China plain	
NQ2050	Niumaohuang	Core collection	Jilin	Northeast China plain	
NQ2051	Bodigao	Core collection	Jilin	Northeast China plain	
NQ2052	Niumaohuang	Core collection	Liaoning	Northeast China plain	
NQ2053	Liushitianhuangcang	Core collection	Liaoning	Northeast China plain	
NQ2054	Nenfengno11	Core collection	Heilongjiang	Northeast China plain	
NQ2055	Dongnong no36	Core collection	Heilongjiang	Northeast China plain	

NQ2056	Heiheixiaohuangdou	Core collection	Heilongjiang	Northeast China plain	
NQ2057	Helongyoutai	Core collection	Jilin	Northeast China plain	
NQ2058	Baichengmoshidou	Core collection	Jilin	Northeast China plain	
NQ2059	Xiaobaiqi	Core collection	Liaoning	Northeast China plain	
NQ2060	Hefeng no25	Core collection	Heilongjiang	Northeast China plain	
NQ2061	Longquandadou	Core collection	Heilongjiang	Northeast China plain	
NQ2062	Xiaolimoshidou	Core collection	Heilongjiang	Northeast China plain	
NQ2063	Qingdou	Core collection	Heilongjiang	Northeast China plain	
NQ2064	Hefeng no24	Core collection	Heilongjiang	Northeast China plain	
NQ2065	Zihua no2	Core collection	Jilin	Northeast China plain	
NQ2066	Chasedou	Core collection	Jilin	Northeast China plain	
NQ2067	TianeDan	Core collection	Liaoning	Northeast China plain	
NQ2068	Daheiqi	Core collection	Liaoning	Northeast China plain	
NQ2070	Yushidou	Core collection	Liaoning	Northeast China plain	
NQ2071	Huangdali	Core collection	Jilin	Northeast China plain	
NQ2072	Chi382	Core collection	Inner-Mongolia	Northen arid and semiarid region	
NQ2073	Zhechengxiaohuangdou	Core collection	Henan	Huang-Huai-Hai plain	Y
NQ2074	Heidou	Core collection	Hebei	Huang-Huai-Hai plain	Y
NQ2075	Huichaxiaohuangdou	Core collection	Sha'anxi	Loess Plateua	
NQ2076	Zaoshuheidou	Core collection	Shandong	Huang-Huai-Hai plain	Y
NQ2077	Gaozuoxuan no1	Core collection	Shandong	Huang-Huai-Hai plain	Y
NQ2078	Pingdinghei	Core collection	Shandong	Huang-Huai-Hai plain	Y
NQ2079	Jidou no7	Core collection	Hebei	Huang-Huai-Hai plain	Y
NQ2080	Maoyandou	Core collection	Hebei	Huang-Huai-Hai plain	Y
NQ2081	Sijiaoqihuangdou	Core collection	Hebei	Huang-Huai-Hai plain	Y

NQ2082	Pingdinghuangdou	Core collection	Shandong	Huang-Huai-Hai plain	Y
NQ2083	Qing no6	Core collection	Shandong	Huang-Huai-Hai plain	Y
NQ2084	Lvcaodou	Core collection	Shandong	Huang-Huai-Hai plain	Y
NQ2085	Chadou	Core collection	Shandong	Huang-Huai-Hai plain	Y
NQ2086	Xichuanjiwohuang	Core collection	Henan	Huang-Huai-Hai plain	Y
NQ2087	Miyangniumaohuang	Core collection	Henan	Huang-Huai-Hai plain	Y
NQ2088	Bo'aihongpizaojiaozi	Core collection	Henan	Huang-Huai-Hai plain	Y
NQ2089	Niumaohuang	Core collection	Sha'anxi	Loess Plateua	
NQ2090	Tongshanqingdadou	Core collection	Jiangsu	Middle-Lower Yangtze plain	Y
NQ2091	Bendidahuangdou	Core collection	Hebei	Huang-Huai-Hai plain	Y
NQ2092	Laoshupi	Core collection	Sha'anxi	Loess Plateua	
NQ2093	Jianghuangdou	Core collection	Sha'anxi	Loess Plateua	
NQ2094	Hualvhuangdou	Core collection	Gansu	Northen arid and semiarid region	
NQ2095	Guanyunhaibaihua	Core collection	Jiangsu	Middle-Lower Yangtze plain	
NQ2096	Huaheihu	Core collection	Hebei	Huang-Huai-Hai plain	Y
NQ2097	Qingdou	Core collection	Hebei	Huang-Huai-Hai plain	Y
NQ2099	Miyangxiaozihuang	Core collection	Henan	Huang-Huai-Hai plain	Y
NQ2100	Binhaidahuangkezijia	Core collection	Jiangsu	Middle-Lower Yangtze plain	Y
NQ2101	Zheng84240-B1	Core collection	Henan	Huang-Huai-Hai plain	Y
NQ2102	Siliyuan	Core collection	Shandong	Huang-Huai-Hai plain	Y
NQ2103	Datian'eDan	Core collection	Shandong	Huang-Huai-Hai plain	Y
NQ2104	Xinyangyangyandou	Core collection	Henan	Huang-Huai-Hai plain	Y
NQ2105	Zheng8516	Core collection	Henan	Huang-Huai-Hai plain	Y
NQ2106	Dabaipi	Core collection	Shandong	Huang-Huai-Hai plain	Y
NQ2107	Diliuhuangdou-2	Core collection	Hebei	Huang-Huai-Hai plain	Y

NQ2108	Shengli no3	Core collection	Shandong	Huang-Huai-Hai plain	Y
NQ2109	Dahuangdou	Core collection	Shandong	Huang-Huai-Hai plain	Y
NQ2110	Xiaomidou	Core collection	Shandong	Huang-Huai-Hai plain	Y
NQ2111	Erliheidou	Core collection	Shandong	Huang-Huai-Hai plain	Y
NQ2112	Peixianxiaoyoudou	Core collection	Jiangsu	Middle-Lower Yangtze plain	
NQ2113	Pixianlayanghuang	Core collection	Jiangsu	Middle-Lower Yangtze plain	Y
NQ2116	Huaiyinchundou	Core collection	Jiangsu	Middle-Lower Yangtze plain	Y
NQ2117	Pixiannianzhuangliuyuexian	Core collection	Jiangsu	Middle-Lower Yangtze plain	Y
NQ2118	Suiningpingdinghuang	Core collection	Jiangsu	Middle-Lower Yangtze plain	Y
NQ2119	Pixiandazihuacao	Core collection	Jiangsu	Middle-Lower Yangtze plain	Y
NQ2120	Pixiansilicao	Core collection	Jiangsu	Middle-Lower Yangtze plain	Y
NQ2121	Shuyangchunheidoubing	Core collection	Jiangsu	Middle-Lower Yangtze plain	Y
NQ2122	Xuanza	Core collection	Yunnan	Yunnan-Guizhou plateau	
NQ2123	Shanzibaihuangdou	Core collection	Hubei	Middle-Lower Yangtze plain	
NQ2124	Cengjialvhuangdou	Core collection	Siquan	Sichuan Basin	
NQ2125	Qiyuehuang	Core collection	Siquan	Sichuan Basin	
NQ2126	Xiaokehuangdou	Core collection	Hubei	Middle-Lower Yangtze plain	
NQ2127	Yizhengdalihuangdou	Core collection	Jiangsu	Middle-Lower Yangtze plain	
NQ2128	0	Core collection	Anhui	Middle-Lower Yangtze plain	
NQ2129	Dimidou	Core collection	Hubei	Middle-Lower Yangtze plain	
NQ2130	Zhongdou no24	Core collection	Hubei	Middle-Lower Yangtze plain	
NQ2131	Chahuangdaidou no1	Core collection	Hubei	Middle-Lower Yangtze plain	
NQ2132	Qianglaiyouheidou	Core collection	Siquan	Sichuan Basin	
NQ2133	Hanyuanbalixioaheidou	Core collection	Siquan	Sichuan Basin	
NQ2134	Lvdouzi	Core collection	Siquan	Sichuan Basin	

NQ2135	Heikewudou	Core collection	Hainan	Southern China
NQ2136	Songzidou	Core collection	Yunnan	Yunnan-Guizhou plateau
NQ2137	Malanzaochadou	Core collection	Yunnan	Yunnan-Guizhou plateau
NQ2138	0	Core collection	Anhui	Middle-Lower Yangtze plain
NQ2139	Cudou	Core collection	Zhejiang	Southern China
NQ2140	Shiyuehuang	Core collection	Siquan	Sichuan Basin
NQ2141	Mayibao	Core collection	Siquan	Sichuan Basin
NQ2142	Lvlanzi	Core collection	Siquan	Sichuan Basin
NQ2143	Zadou-6	Core collection	Guizhou	Yunnan-Guizhou plateau
NQ2144	Wujiangwuyueniumaohuang	Core collection	Jiangsu	Middle-Lower Yangtze plain
NQ2145	Jinghuang35yi	Core collection	Hubei	Middle-Lower Yangtze plain
NQ2146	Shangraobayuebai	Core collection	Jiangxi	Middle-Lower Yangtze plain
NQ2147	Dantuxiaowujia	Core collection	Jiangsu	Middle-Lower Yangtze plain
NQ2148	82-16	Core collection	Hubei	Middle-Lower Yangtze plain
NQ2149	Chihuangdou-1	Core collection	Hubei	Middle-Lower Yangtze plain
NQ2150	Huameidou	Core collection	Hubei	Middle-Lower Yangtze plain
NQ2151	Wuyanwo	Core collection	Siquan	Sichuan Basin
NQ2152	Yangyandou	Core collection	Yunnan	Yunnan-Guizhou plateau
NQ2153	Dawudou	Core collection	Guangxi	Southern China
NQ2154	Jiangehualinjiwodou	Core collection	Siquan	Sichuan Basin
NQ2155	Mashanrenfenghuangdou	Core collection	Guangxi	Southern China
NQ2156	Shuguanghuangdou	Core collection	Hubei	Middle-Lower Yangtze plain
NQ2157	Qiyuehuang-1	Core collection	Guizhou	Yunnan-Guizhou plateau
NQ2159	74-424	Core collection	Hubei	Middle-Lower Yangtze plain
NQ2160	Chihuangdou no2	Core collection	Hubei	Middle-Lower Yangtze plain

NQ2161	Taixingheidou	Core collection	Jiangsu	Middle-Lower Yangtze plain
NQ2162	Hengfengwudou	Core collection	Jiangxi	Middle-Lower Yangtze plain
NQ2163	Taixingaijiaohong	Core collection	Jiangsu	Middle-Lower Yangtze plain
NQ2164	Nidou	Core collection	Hubei	Middle-Lower Yangtze plain
NQ2165	77-391-1	Core collection	Jiangsu	Middle-Lower Yangtze plain
NQ2166	Shifangluosidou	Core collection	Siquan	Sichuan Basin
NQ2167	Wuyuehuang	Core collection	Jiangxi	Middle-Lower Yangtze plain
NQ2168	Duchuangwudou	Core collection	Jiangxi	Middle-Lower Yangtze plain
NQ2169	Zaowudou	Core collection	Jiangxi	Middle-Lower Yangtze plain
NQ2170	2340322	Core collection	Siquan	Sichuan Basin
NQ2171	Xiandou no7	Core collection	Siquan	Sichuan Basin
NQ2172	84-70	Core collection	Hubei	Middle-Lower Yangtze plain
NQ2173	Xiaobaimao	Core collection	Siquan	Sichuan Basin
NQ2174	Bazhongtianyindou	Core collection	Siquan	Sichuan Basin
NQ2175	Quxianbayuehuang	Core collection	Siquan	Sichuan Basin
NQ2176	Zizhongliuyuezao	Core collection	Siquan	Sichuan Basin
NQ2177	Pixianxiaohuangdou	Core collection	Siquan	Sichuan Basin
NQ2178	Honghuliuyuebao	Core collection	Hubei	Middle-Lower Yangtze plain
NQ2179	Huasedou	Core collection	Hubei	Middle-Lower Yangtze plain
NQ2180	Xiangdou no4	Core collection	Hunan	Middle-Lower Yangtze plain
NQ2181	Jianweiquanshuidou	Core collection	Siquan	Sichuan Basin
NQ2182	Pengshanhuangkezi-3	Core collection	Siquan	Sichuan Basin
NQ2183	Shaxindou	Core collection	Jiangxi	Middle-Lower Yangtze plain
NQ2184	Ruijinqingpidou	Core collection	Jiangxi	Middle-Lower Yangtze plain
NQ2185	Shaxianwudou	Core collection	Fujian	Middle-Lower Yangtze plain

NQ2186	Huangmaodou	Core collection	Hunan	Middle-Lower Yangtze plain
NQ2187	Zaoshumaolianqing	Core collection	Zhejiang	Southern China
NQ2188	Shaxianqingdou	Core collection	Fujian	Middle-Lower Yangtze plain
NQ2189	Baiqiu no1	Core collection	Fujian	Middle-Lower Yangtze plain
NQ2190	Ai'shengnidou	Core collection	Hunan	Middle-Lower Yangtze plain
NQ2191	Zhao'anqiudou	Core collection	Fujian	Middle-Lower Yangtze plain
NQ2192	Xinyudaliqing	Core collection	Jiangxi	Middle-Lower Yangtze plain
NQ2193	Changshanidou	Core collection	Hunan	Middle-Lower Yangtze plain
NQ2194	Hongzhudou	Core collection	Hunan	Middle-Lower Yangtze plain
NQ2195	Dahuangzhu	Core collection	Jiangxi	Middle-Lower Yangtze plain
NQ2196	Yangshanqingdou	Core collection	Guangdong	Middle-Lower Yangtze plain
NQ2197	En'pingqingdou	Core collection	Guangdong	Middle-Lower Yangtze plain
NQ2198	Madaiqingdou-3	Core collection	Guangdong	Middle-Lower Yangtze plain
NQ2199	Dongshanbaimadou	Core collection	Fujian	Middle-Lower Yangtze plain
NQ2200	Daqingren	Core collection	Fujian	Middle-Lower Yangtze plain
NQ2201	Pudou451	Core collection	Fujian	Middle-Lower Yangtze plain
NQ2202	Dalihuang	Core collection	Fujian	Middle-Lower Yangtze plain
NQ2203	Tong'anzihongdou	Core collection	Fujian	Middle-Lower Yangtze plain
NQ2204	Baimaodou	Core collection	Guizhou	Yunnan-Guizhou plateau
NQ2205	Dabaimaodou	Core collection	Guangdong	Middle-Lower Yangtze plain
NQ2206	Longchuanhuangniumao	Core collection	Guangdong	Middle-Lower Yangtze plain
NQ2207	Lianjiangpohuangdou	Core collection	Guangdong	Middle-Lower Yangtze plain
NQ2208	Erjizaodou-2	Core collection	Guizhou	Yunnan-Guizhou plateau
NQ2209	Shamentengzidou	Core collection	Fujian	Middle-Lower Yangtze plain
NQ2210	Dahuangdou-1	Core collection	Guizhou	Yunnan-Guizhou plateau

NQ2211	Qingyuandaqingdou	Core collection	Guangdong	Middle-Lower Yangtze plain	
NQ2212	Dahuangdou-2	Core collection	Guangdong	Middle-Lower Yangtze plain	
NQ2213	Madaiqingdou-2	Core collection	Guangdong	Middle-Lower Yangtze plain	
NQ2214	Xihuangdou9	Core collection	Guizhou	Yunnan-Guizhou plateau	
NQ2215	Zaojiaodou	Core collection	Guizhou	Yunnan-Guizhou plateau	
NQ2216	Xihuangdou-8	Core collection	Guizhou	Yunnan-Guizhou plateau	
NQ2217	Zaohuangdou	Core collection	Guizhou	Yunnan-Guizhou plateau	
NQ2218	Xiaoheidou	Core collection	Shanxi	Loess Plateua	
NQ2221	Huangqi	Core collection	Liaoning	Northeast China plain	
NQ2222	Niupihuangdou	Core collection	Sha'anxi	Loess Plateua	
NQ2223	Dahuangdou	Core collection	Sha'anxi	Loess Plateua	
NQ2224	Pixianhongmaoyou	Core collection	Jiangsu	Middle-Lower Yangtze plain	Y
NQ2225	Dahuadou	Core collection	Siquan	Sichuan Basin	
NQ2226	Huangdou	Core collection	Yunnan	Yunnan-Guizhou plateau	
NQ2227	Qionglaihuamhmaozi	Core collection	Siquan	Sichuan Basin	
NQ2228	Donghuangdou1	Core collection	Siquan	Sichuan Basin	
NQ2229	Baimaozaodouzi	Core collection	Siquan	Sichuan Basin	
NQ2230	Yantianqingpidou	Core collection	Jiangxi	Middle-Lower Yangtze plain	
NQ2232	Touxinlv	Core collection	Siquan	Sichuan Basin	
NQ2234	Yizhangliuyuehuang	Core collection	Hunan	Middle-Lower Yangtze plain	
NQ2235	Suiningfengtaijiangsedou	Core collection	Siquan	Sichuan Basin	

TABLE S2| Descriptive statistics of phytate-phosphorus (P) concentration of 256 soybean genotypes commonly cultivated in China.

Province	Region	Phytate P mg g^{-1}	CV%
Gansu (N=3)	Northern arid and semiarid region	2.31 E	35.35
Shaanxi (N=10)	Loess Plateua	2.32E	30.79
Shandong (N=18)	Huang-Huai-Hai plain	2.39E	47.40
Henan (N=9)	Huang-Huai-Hai plain	2.46DE	28.00
Guangdong (N=9)	Middle-Lower Yangtze plain	2.47DE	51.79
Inner Mongoliya (N=3)	Northern arid and semiarid region	2.50DE	31.79
Beijing (N=1)	Northern arid and semiarid region	2.56DE	–
Shanxi (N=20)	Loess Plateua	2.59DE	24.16
Liaoning (N=9)	Northeast China plain	2.61 DE	21.74
Fujian (N=10)	Middle-Lower Yangtze plain	2.65CDE	28.64
Hebei (N=12)	Northern arid and semiarid region	2.72CDE	30.02
Hunan (N=6)	Middle-Lower Yangtze plain	2.76CDE	32.37
Guizhou (N=10)	Yunnan-Guizhou plateau	2.80CDE	34.15
Jilin (N=14)	Northeast China plain	2.88BCDE	24.48
Xinjiang (N=2)	Northern arid and semiarid region	2.92BCDE	9.51
HuBei (N=18)	Middle-Lower Yangtze plain	2.93BCDE	27.25
Yunnnan (N=6)	Yunnan-Guizhou plateau	2.96BCDE	26.79
Heilongjiang (N=25)	Northeast China plain	2.99BCDE	22.63
Jiangxi (N=10)	Middle-Lower Yangtze plain	3.02 BCDE	20.70
Sichuan (N=30)	Sichuan Basin	3.07BCDE	24.37
Ningxia (N=1)	Northern arid and semiarid region	3.18 BCDE	–
Jiangsu (N=16)	Middle-Lower Yangtze plain	3.33BCDE	31.75
Guangxi (N=2)	Southern China	3.48 BCD	0.17
Zhejiang (N=2)	Southern China	3.62BC	3.98
Anhui (N=3)	Middle-Lower Yangtze plain	3.83B	12.55
Hainan (N=1)	Southern China	5.08 A	–

TABLE S3| Variable scores of nine plants traits in 43 soybean genotypes and proportion of variation of each component. The largest variable loading scores for each traits in the first three components are in bold.

	PC1	PC2	PC3
Seed phytate-P concentration	-0.06	0.04	**0.60**
Shoot dry weight	0.24	**-0.49**	**0.44**
Root dry weight	**0.47**	0.04	0.11
Root/ shoot_ratio	0.35	**0.45**	-0.26
Carboxylate amount per root DW	0.21	-0.13	0.23
Total root length	**0.47**	0.12	0.03
Root surface area	**0.49**	0.12	0.09
Acid phospatates	-0.07	**0.58**	**0.36**
Rhizosheath soil dry weight	0.28	**-0.41**	-0.41
